# Spinal fusion surgery - the need to follow the ‘BRAN’ toolkit (benefits, risks, alternatives, nothing): a case report

**DOI:** 10.1093/jscr/rjac431

**Published:** 2022-09-20

**Authors:** George Ampat, Jemima S George, Abigail L Clynch, Jonathan M G Sims

**Affiliations:** School of Medicine, University of Liverpool, Liverpool, UK; Darlington Memorial Hospital, Darlington, UK; School of Medicine, University of Liverpool, Liverpool, UK; Talita Cumi Ltd., Southport, UK

## Abstract

Failed back surgery syndrome is defined as increased or persistent pain following spinal surgery. Despite a relatively high incidence of failed back surgery syndrome (20%), patients may not be counselled regarding this complication pre-operatively. The Academy of Medical Royal Colleges has provided the Benefits, Risks, Alternatives and doing Nothing Toolkit to guide clinical discussions during the consent process. A 46-year-old female experiencing chronic lower back pain since 2003 suffered an exacerbation in 2015. Imaging identified non-compressive disc bulges***.*** She was not put through the low back pain pathway as recommended by NICE and underwent spinal fusion in 2017. She continues to experience severe pain 54 months postsurgery. When considering spinal surgery, the risk of failed back surgery syndrome should be discussed with patients. Both clinicians and patients can use the BRAN toolkit to ensure open and transparent discussion prior to any intervention.

## INTRODUCTION

Low back pain is the leading cause of years lived with disability [1]. Surgical intervention to treat lower back pain is on the rise, with spinal fusion rates increasing by 62.3% between 2004 and 2015 [2]. This increase in spinal fusion rates has occurred despite evidence showing no clinically significant difference between fusion surgery and non-operative management for lower back pain [3].

Complications and negligence are high in spinal surgery, with £100 million of the NHS budget (one-third of the total spinal surgery budget) spent annually on litigation [4]. Failed back surgery syndrome (FBSS), a potential contributor to litigation in orthopedic surgery, affects 20–40% of patients [5]. FBSS is defined as persistent severe pain even following surgical intervention [5]. Compared with other chronic conditions (e.g. rheumatoid arthritis), FBSS results in lower quality of life (QoL) and higher rates of disability and unemployment [5, 6].

## CASE REPORT

A 46-year-old female had experienced chronic lower back pain since her late twenties. She suffered an acute episode of increased back pain in April 2015 and a further exacerbation following a side-impact collision in October 2015.

**Figure 1:**
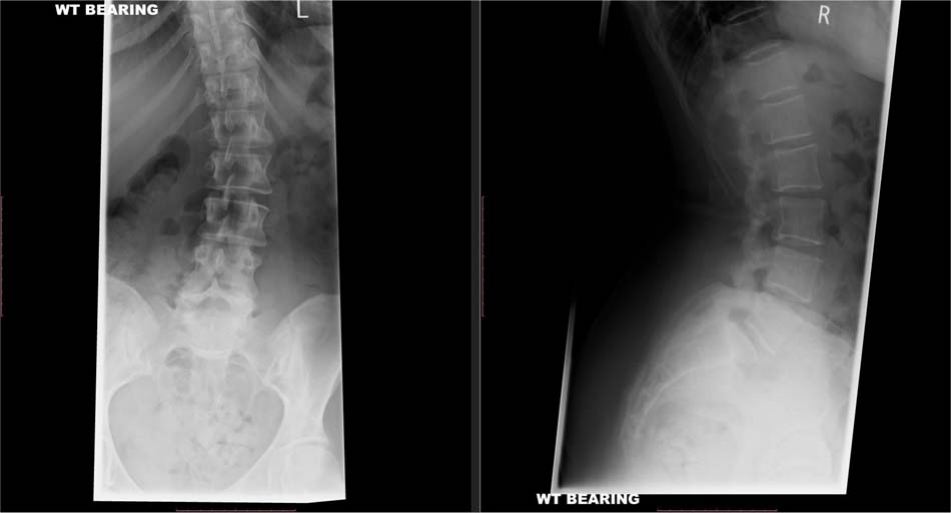
Preoperative X-ray of the lumbar spine performed in December 2015, confirming small idiopathic scoliosis in the lumbar spine.

X-rays of the lumbar spine (Fig. 1) and pelvis performed in December 2015 showed a small idiopathic scoliosis in the lumbar spine. A magnetic resonance imaging (MRI) scan was performed in February 2016, which showed non-compressive disc degeneration and disc bulge at L4/5 and L5/S1. She was referred to physiotherapy for rehabilitation.

Medications prescribed included codeine, naproxen, amitriptyline and gabapentin. The patient stopped taking gabapentin as she began suffering from psychological issues, including depression and irritability. She trialled transcutaneous electrical nerve stimulation (TENS) in April 2016 and underwent a left L5 nerve block injection in October 2016.

**Figure 2:**
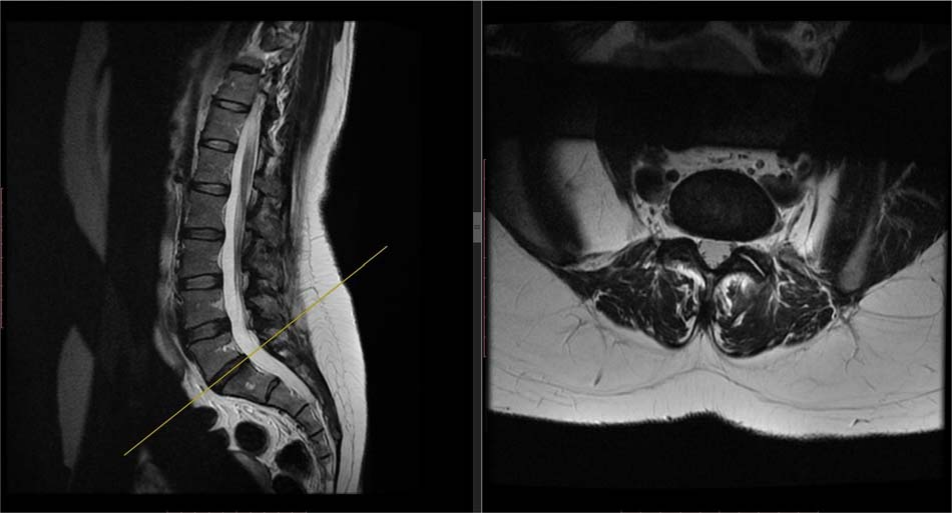
Preoperative MRI of the lumbar spine performed in December 2016. The MRI shows no neurological compression.

A repeat MRI scan (Fig. 2) performed in December 2016 did not show any interval changes as compared to the scan performed in February 2016. The patient presented elsewhere and was recommended for spinal fusion surgery. A posterior pedicle screw instrumentation with rods and a posterior interbody cage procedure was performed elsewhere in January 2017.

**Figure 3:**
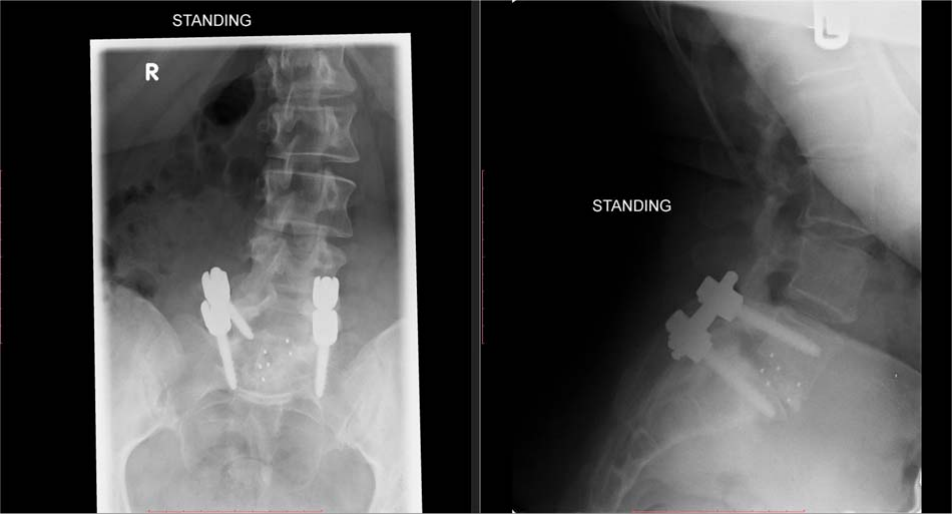
Postoperative X-ray of the lumbar spine performed in January 2017, showing the implants in place.

**Figure 4:**
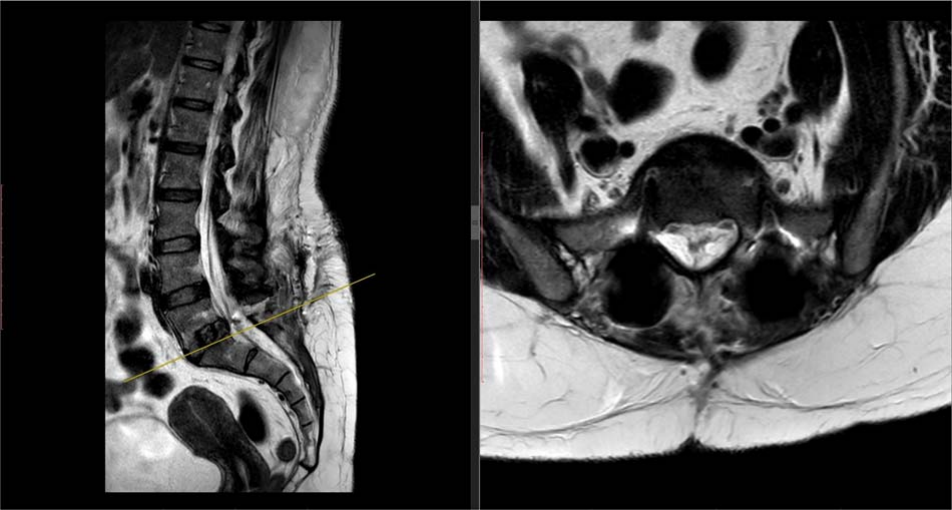
Postoperative MRI of the lumbar spine performed in January 2017. Interference from the metallic pedicular screws limited the scans clarity.

Postoperatively, the patient developed severe radiculopathy down the left leg and required opiates. The patient could not move the left leg and was unable to mobilize. X-rays showed the implants were in position (Fig. 3), but the information from a fresh MRI scan was limited due to metal artefact (Fig. 4). With ongoing, unrelenting pain, she was returned to theatre on the fourth postoperative date, and the wound was re-explored. Intraoperatively, no dural tear was identified, the nerve roots were free, and the screws appeared in position. She was then discharged home after ten nights in hospital, instead of the standard three nights that would be common practice.

In the ensuing months, the patient continued to experience severe pain, and imaging investigations did not identify any cause for her symptoms. Her pain scores on a scale of 0 to 10 remain at 8/10, 54 months following the surgical intervention. She continues to take naproxen and tramadol for the pain. She has also been prescribed pramipexole for restless legs. The patient initially managed to return to work on a part-time basis. However, in September 2020, she had to give up her employment as a social worker due to persistent pain. Clinical examination at the last follow-up revealed persistent numbness in the left L5 dermatome and weakness of the left L5 myotome. The numbness and weakness of the left leg were not present prior to the surgical intervention.

## DISCUSSION

Spinal surgery for low back pain is a complex procedure that requires extensive consideration by both clinician and patient, coming with an array of complications and the potential to worsen symptoms and disability, as seen in FBSS [6]. FBSS can contribute to significantly reduced QoL in patients [6], and treatment options are limited. FBSS has the highest treatment cost implications (€1802 per annum) compared with other chronic pain conditions, including rheumatoid arthritis, osteoarthritis and fibromyalgia (€183–€1261 per annum) [7].

At least 1 in 5 spinal surgeries results in FBSS [5]. To our knowledge, the above patient was not aware of FBSS as a complication prior to surgery. It is important that patients should be counselled that there is a 1 in 5 risk of persistent severe pain following spinal fusion and for which treatment options may be limited. Providing this information would be in line with the Montgomery v Lanarkshire Health Board Supreme Court ruling [8]. The General Medical Council also provides clear recommendations regarding consent [9].

The National Low Back and Radicular Pain Pathway [10] provides clear guidance on how patients should be treated. Though the above patient was referred to physiotherapy, she did not have Comprehensive Core Therapy or a Comprehensive Combined Physical and Psychological Programme (CPPP), as suggested in boxes 10 and 12, respectively, of the pathway [10].

It is possible that the patient might have had persistent spinal pain even in the absence of surgical intervention. However, if the pathway had been followed, it is possible that she might have obtained relief without the need for an expensive and irreversible surgical procedure. Surgery should not be performed just because it can be done.

Choosing Wisely UK, hosted at the Academy of Medical Royal Colleges, is part of a global initiative aimed at improving conversations between patients and their health providers. They have provided a helpful toolkit to aid decision-making, BRAN, which stands for Benefits, Risks, Alternatives and what if Nothing is done [11]. They have also provided intervention-specific leaflets for spinal surgical fusion, which clearly state, ‘Spinal fusion surgery is not helpful for mechanical lower back pain and may make the pain worse’ [12].

## CONCLUSION

The low back pain pathway, as recommended by NICE, should be followed in treating patients with low back and radicular pain. If surgical intervention is considered, then the BRAN toolkit, as recommended by The Academy of Medical Royal College, should be used to guide the consenting process. Patients should be counselled about the real risk of FBSS or persistent spinal pain following surgery.
